# Refinements and Extensions of Ziv’s Model of Perfect Secrecy for Individual Sequences

**DOI:** 10.3390/e26060503

**Published:** 2024-06-09

**Authors:** Neri Merhav

**Affiliations:** The Viterbi Faculty of Electrical and Computer Engineering, Technion—Israel Institute of Technology, Technion City, Haifa 3200003, Israel; merhav@technion.ac.il; Tel.: +972-4-8294737

**Keywords:** encryption, information security, perfect secrecy, finite-state machines, universal coding, Lempel–Ziv algorithm

## Abstract

We refine and extend Ziv’s model and results regarding perfectly secure encryption of individual sequences. According to this model, the encrypter and the legitimate decrypter share a common secret key that is not shared with the unauthorized eavesdropper. The eavesdropper is aware of the encryption scheme and has some prior knowledge concerning the individual plaintext source sequence. This prior knowledge, combined with the cryptogram, is harnessed by the eavesdropper, who implements a finite-state machine as a mechanism for accepting or rejecting attempted guesses of the plaintext source. The encryption is considered perfectly secure if the cryptogram does not provide any new information to the eavesdropper that may enhance their knowledge concerning the plaintext beyond their prior knowledge. Ziv has shown that the key rate needed for perfect secrecy is essentially lower bounded by the finite-state compressibility of the plaintext sequence, a bound that is clearly asymptotically attained through Lempel–Ziv compression followed by one-time pad encryption. In this work, we consider some more general classes of finite-state eavesdroppers and derive the respective lower bounds on the key rates needed for perfect secrecy. These bounds are tighter and more refined than Ziv’s bound, and they are attained using encryption schemes that are based on different universal lossless compression schemes. We also extend our findings to the case where side information is available to the eavesdropper and the legitimate decrypter but may or may not be available to the encrypter.

## 1. Introduction

Theoretical frameworks focusing on individual sequences and finite-state encoders and decoders have undergone extensive exploration, diverging from the conventional probabilistic paradigm used in modeling sources and channels. This divergence has been particularly evident across various information-theoretic domains, such as data compression [1,2,3,4,5,6,7], source/channel simulation [8,9], classification [10], prediction [11,12,13,14], denoising [15], and even channel coding [16,17,18]. These references merely scratch the surface of a vast body of literature. Conversely, the realm of information-theoretic security, from Shannon’s seminal contribution [19] to more contemporary research [20,21,22,23,24], remains almost totally entrenched in the probabilistic framework. While these works represent a mere fraction of the extensive literature, they exemplify the nearly exclusive reliance on probabilistic models within this field.

To the best of the author’s knowledge, there are only two exceptions to this prevailing paradigm, documented in an unpublished memorandum by Ziv [25] and a subsequent work [26]. Ziv’s memorandum presents a unique approach wherein the plaintext source, to be encrypted using a secret key, is treated as an individual sequence. The encrypter is modeled as a general block encoder, while the eavesdropper employs a finite-state machine (FSM) as a message discriminator. The memorandum postulates that the eavesdropper possesses certain prior knowledge about the plaintext, expressed as a set of “acceptable messages”, hereafter referred to as the *acceptance set*. In other words, before observing the cryptogram, the eavesdropper’s uncertainty about the plaintext sequence is that it could be any member of this set of acceptable messages.

This assumption about the prior knowledge available to the eavesdropper is fairly realistic in real life. Consider, for example, the case where the plaintext alphabet is the Latin alphabet, but the eavesdropper knows that the plaintext must be a piece of text in the Italian language. In this case, their prior knowledge allows them to reject every candidate string of symbols that includes the letters ‘j’, ‘k’, ‘w’, ‘x’, and ‘y’, which are not used in Italian. Another example, which is common to English and some other languages, is that the letter ‘q’ must be followed by ‘u’. In the same spirit, some additional rules of grammar can be invoked, like limitations on the number of successive consonants (or vowels) in a word, limitations on the length of a word, and so on.

Now, according to Ziv’s approach, perfectly secure encryption amounts to a situation where the presence of the cryptogram does not reduce the uncertainty associated with the acceptance set. In other words, having intercepted the cryptogram, the eavesdropper learns nothing about the plaintext that they did not know before. The size of the acceptance set can be thought of as a quantifier of the level of uncertainty: a larger set implies greater uncertainty. The aforementioned FSM is used to discriminate between acceptable and unacceptable strings of plaintext symbols that can be obtained by examining various key bit sequences. Accordingly, perfect security amounts to maintaining the size of the acceptance set unchanged, and consequently, the uncertainty level, in the presence of the cryptogram. The principal finding in Ziv’s work is that the asymptotic key rate required for perfectly secure encryption, according to this definition, cannot be lower (up to asymptotically vanishing terms) than the Lempel–Ziv (LZ) complexity of the plaintext source [7]. Clearly, this lower bound is asymptotically achieved by employing one-time pad encryption (that is, bit-by-bit XOR with key bits) of the bit-stream obtained from LZ data compression of the plaintext source, mirroring Shannon’s classical probabilistic result, which asserts that the minimum required key rate equals the entropy rate of the source.

In the subsequent work [26], the concept of perfect secrecy for individual sequences was approached differently. Instead of a finite-state eavesdropper with predefined knowledge, it was assumed that the encrypter could be realized by an FSM, which is sequentially fed by the plaintext source and random key bits. A notion of “finite-state encryptability” was introduced (in the spirit of the analogous finite-state compressibility in [7]), which designates the minimum key rate that must be consumed by any finite-state encrypter, such that the probability law of the cryptogram would be independent of the plaintext input, and hence be perfectly secure. Among the main results in [26], it was asserted and proven that the finite-state encryptability of an individual sequence is essentially bounded from below by its finite-state compressibility, a bound that is once again attained asymptotically through LZ compression followed by one-time pad encryption.

In this work, we revisit Ziv’s approach to perfect secrecy for individual sequences [25]. After presenting his paradigm in detail, we proceed to refine and generalize his findings in certain aspects. First, we consider several more general classes of finite-state discriminators that can be employed by the eavesdropper. These will lead to tight lower bounds on the minimum key rate to be consumed by the encrypter, which will be matched by encryption schemes that are based on some other universal data compression schemes. The resulting gaps between the lower bounds and the corresponding upper bounds (i.e., the redundancy rates) would converge faster. Among these more general classes of finite-state machines, we will consider finite-state machines that are equipped with counters, as well as periodically time-varying finite-state machines with counters. Another direction of generalizing Ziv’s findings is the incorporation of side information (SI) available to both the eavesdropper and the legitimate decrypter, but which may or may not be available to the encrypter.

The outline of this article is as follows. In Section 2, we formulate the model setting, establish the notations, and provide a more detailed background on Ziv’s model and results in [25]. In Section 3, which is the main section of this article, we present the refinements and extensions to other types of FSMs, including FSMs with counters (Section 3.1), shift-register FSMs with counters (Section 3.2), and periodically time-varying FSMs with counters (Section 3.3). Finally, in Section 4, we further extend some of our findings to the case where SI is available to both the legitimate decrypter and the eavesdropper but not necessarily to the encrypter.

## 2. Formulation, Notations, and Background

Consider the following version of Shannon’s cipher system model, adapted to the encryption of individual sequences, as proposed by Ziv [25]. An individual (deterministic) plaintext sequence, $x=(x_{0},\ldots ,x_{n-1})$ (*n*—positive integer), is encrypted using a random key *K*, whose entropy is H(K), to generate a cryptogram, W=T(x,K), where the mapping T(·,K) is invertible given *K*, namely ***x*** can be reconstructed by the legitimate decoder, who has access to *K*, by applying the inverse function, $x=T^{-1}\left(W,K\right)$. The plaintext symbols, $x_{i}$, i=0,1,2,…,n−1, take on values in a finite alphabet, X, of size α. Thus, ***x*** is a member of $X^{n}$, the *n*-th Cartesian power of X, whose cardinality is $\alpha^{n}$. Without essential loss of generality, we assume that *K* is a uniformly distributed random variable taking on values in a set K, whose cardinality is $2^{H(K)}$. Sometimes, it may be convenient to consider K as the set $\left\{0,1,\ldots ,2^{H(K)}-1\right\}$. A specific realization of the key, *K*, is denoted by *k*.

An eavesdropper who knows the mapping, *T*, but not the realization of the key, *K*, is in the quest of learning as much as possible about ***x*** upon observing *W*. It is assumed that the eavesdropper also has some prior knowledge about ***x***, even before observing *W*. In particular, the eavesdropper knows that the plaintext source string ***x*** must be a member of a certain subset of $X^{n}$, denoted as $A_{n}$, which is referred to as the *acceptance set*.

Ziv models the eavesdropper by a cascade of a guessing decrypter and a finite-state message discriminator, which work together as follows. At each step, the eavesdropper examines a certain key, k∈K, by generating an estimated plaintext, $\hat{x}=T^{-1}\left(W,k\right)$, and then feeding $\hat{x}$ into the message discriminator to examine whether $\hat{x}\in A_{n}$. If the answer is affirmative, $\hat{x}$ is accepted as a candidate; otherwise, it is rejected. Upon completing this step, the eavesdropper moves on to the next key, k+1, and repeats the same process. The message discriminator is modeled as a finite-state machine, which implements the following recursive equations for i=0,1,2,…,n−1:

(1) $$\begin{array}{ccc} u_{i} & = & f(z_{i},{\hat{x}}_{i}) \end{array}$$

(2) $$\begin{array}{ccc} z_{i+1} & = & g(z_{i},{\hat{x}}_{i}), \end{array}$$

where $z_{0},z_{1},z_{2},\ldots ,z_{n-1}$ is a sequence of states; $z_{i}\in S$, i=0,1,2,…,n−1; S is a set of *s* states (and with the initial state, $z_{0}$, as a fixed member of S); $u_{0},u_{1},\ldots ,u_{n-1}$ is a binary output sequence that indicates acceptance or rejection; f:S×X→{0,1} is the output function; and g:S×X→S is the next-state function. If $u_{0},u_{1},u_{2},\ldots ,u_{n-1}$ is the all-zero sequence, $\hat{x}$ is accepted, namely $\hat{x}\in A_{n}$; otherwise, as soon as $u_{i}=1$ for some 0≤i≤n−1, $\hat{x}$ is rejected. In other words, $A_{n}$ is defined as the set of all $\left\{\hat{x}\right\}$, for which the response of the finite-state discriminator is the all-zero sequence, u=(0,0,…,0).

**Example** **1.** *Let X={0,1}, and suppose that the membership of **x** in $A_{n}$ forbids the appearance of more than two successive zeroes. Then, a simple discriminator can detect a violation of this rule using the finite-state machine defined by S={0,1,2} and*

(3) $$\begin{array}{ccc} g(z,\hat{x}) & = & \left\{\begin{array}{cc} (z+1)\;\mathrm{mod}\;3 & \hat{x}=0\\ 0 & \hat{x}=1 \end{array}\right. \end{array}$$

(4) $$\begin{array}{ccc} f(z,\hat{x}) & = & \left\{\begin{array}{cc} 1 & z=2\;and\;\hat{x}=0\\ 0 & otherwise \end{array}\right. \end{array}$$

*with the initial state being $z_{0}=0$. More generally, consider the set of binary sequences that comply with the so-called (d,k) constraints, well known in the literature on magnetic recording (see, e.g., [27] and the references therein). These are binary sequences, where the runs of successive zeroes must be of length at least d and at most k, where d and k (not to be confused with the notation of the encryption key) are positive integers with d≤k. We return to this example later.*

Ziv defines perfect secrecy for individual sequences as a situation where, even upon observing *W*, the eavesdropper’s uncertainty about ***x*** is not reduced. In mathematical terms, let us denote

(5) $$T^{-1}\left(W\right)\overset{▵}{=}\left\{T^{-1}\left(W,k\right),\;k\in K\right\},$$

and

(6) $$A_{n}\left(W\right)\overset{▵}{=}A_{n}⋂T^{-1}\left(W\right),$$

and then perfect secrecy is defined as a situation where

(7) $$A_{n}\left(W\right)=A_{n},$$

or equivalently,

(8) $$A_{n}\subseteq T^{-1}\left(W\right).$$

To demonstrate these concepts, consider the following example.

**Example** **2.** *Let n=4, X={0,1}, x=(1111), $2^{H(K)}=8$, and k=(1111). Then, for a one-time pad encrypter,*

(9) W=T(x,k)=x⊕k=(1111)⊕(1111)=(0000),

*where *⊕* denotes bit-wise XOR (modulo 2 addition). Let the set K of all eight possible key strings be given by*

(10) $$K=\left\{\begin{array}{c} 1111\\ 1000\\ 1100\\ 1001\\ 0000\\ 0111\\ 0011\\ 0110 \end{array}\right\}.$$

*Obviously, the decryption is given by $T^{-1}\left(W,k\right)=W⊕k$. Since W=(0000), then $T^{-1}\left(W\right)=K$. Following Example 1, suppose that $A_{4}$ is the set of all binary vectors of length n=4, which do not contain runs of more than two zeroes. There are only three binary vectors of length 4 that contain a succession of more than 2 (i.e., 3 or 4) zeroes, namely (0000), (1000), and (0001). Thus, $|A_{4}|=2^{4}-3=13$. On the other hand,*

(11) $$|A_{4}\left(W\right)|=|A_{4}⋂T^{-1}\left(W\right)|\leq |T^{-1}\left(W\right)|=|K|=8<13,$$

*where the notation |E| for a generic finite set E denotes its cardinality. This means that this encryption system is not perfectly secure. The reason for this is that the key space, K, is not large enough.*

Clearly, in the quest to minimize H(K) without compromising perfect secrecy, the optimal approach is to design the encrypter in such a way that

(12) $$T^{-1}\left(W\right)=A_{n},$$

for every cryptogram *W* that can possibly be obtained from some combination of ***x*** and *k*. Conceptually, this can be obtained by mapping $A_{n}$ to the set of all binary sequences of length H(K) utilizing a fixed-rate data compression scheme and applying one-time pad encryption to the compressed sequence. Here, and throughout the following discussion, we neglect integer length constraints associated with large numbers, so H(K) is assumed to be an integer without essential loss of generality and optimality.

**Remark** **1.** *For readers familiar with the concepts and the terminology of coding for systems with (d,k) constraints (other readers may skip this remark without loss of continuity), it is insightful to revisit the second part of Example 1: If $A_{n}$ is the set of binary n-sequences that satisfy a certain (d,k) constraint, then optimal encryption for $A_{n}$ pertains to compression using the inverse mapping of a channel encoder for the same (d,k) constraint, namely the corresponding channel decoder, which is followed by one-time pad encryption. The minimum key rate needed is then equal to the capacity of the constrained system, which can be calculated either algebraically, as the logarithm of the Perron–Frobenius eigenvalue of the state adjacency matrix of the state transition diagram, or probabilistically, as the maximum entropy among all stationary Markov chains that are supported by the corresponding state transition graph [27].*

Ziv’s main result in [25] was that for a finite-state discriminator, if $x\in A_{n}$, the cardinality of $A_{n}$ cannot be exponentially smaller than $2^{LZ(x)}$ (see Appendix A for the proof), where LZ(x) is the length (in bits) of the compressed version of ***x***, using the 1978 version of the Lempel–Ziv algorithm (the LZ78 algorithm) [7]. Therefore, the key rate needed to completely encrypt all members of $A_{n}$ is lower bounded by

(13) $$\begin{array}{cc} R & \overset{▵}{=}\frac{H(K)}{n}\\ & =\frac{\log|T^{-1}\left(W\right)|}{n}\\ & \geq \frac{\log|A_{n}|}{n}\\ & \geq \frac{LZ(x)}{n}-\epsilon_{n}, \end{array}$$

where $\epsilon_{n}$ is a positive sequence tending to zero as n→∞ at the rate of $\frac{\log(\log\;n)}{\log\;n}$. The first inequality of (13) follows from (8). Obviously, this bound is essentially attained through LZ78 compression of ***x***, followed by one-time pad encryption using LZ(x) key bits. As can be seen, the gap, $\epsilon_{n}$, between the upper bound and the lower bound on *R* is $O\left(\frac{\log(\log\;n)}{\log\;n}\right)$, which tends to zero rather slowly.

**Remark** **2.** *For an infinite sequence $x=x_{0},x_{1},x_{2},\ldots$, asymptotic results were obtained in [25] through a two-stage limit process: First, consider a finite sequence of total length m·n, which is divided into m non-overlapping n-blocks. The mechanism described above is applied to each n-block separately. The asymptotic minimum key rate is obtained by taking a double limit superior, first for m→∞ for a given n, and then for n→∞. In this work, we consider a similar double limit, although we do not explicitly mention it on every relevant occasion. Instead, we focus on the behavior of a single n-block.*

## 3. More General Finite-State Discriminators

This section, which is the main section of this article, is devoted to describing several more general classes of finite-state discriminators, along with derivations of their respective more refined bounds.

### 3.1. FSMs with Counters

While Ziv’s model for a finite-state discriminator is adequate for rejecting sequences with certain forbidden patterns (like a succession of more than two zeroes in the above examples), it is not sufficient to handle situations like the following. Suppose that the encrypter applies a universal lossless compression algorithm for memoryless sources, followed by one-time pad encryption. Suppose also that the universal compression scheme is a two-part code, where the first part encodes the index of the type class of ***x***, using a number of bits that is proportional to log n, and the second part represents the index of ***x*** within the type class, assuming that the encoder and the decoder have agreed on some ordering ahead of time. In this case, the length of the cryptogram (in bits), which is equal to the length of the compressed data, is about

(14) $$L=H\left(K\right)\approx n\hat{H}\left(x\right)+\frac{\alpha -1}{2}\cdot \log\;n,$$

where $\hat{H}\left(x\right)$ is the empirical entropy of ***x*** (see, e.g., [3] and the references therein). The eavesdropper, being aware of the encryption scheme, observes the length *L* of the cryptogram and immediately concludes that ***x*** must be a sequence whose empirical entropy is

(15) $$H_{0}=\frac{1}{n}\cdot \left(L-\frac{\alpha -1}{2}\cdot \log n\right).$$

In other words, in this case,

(16) $$A_{n}\left(W\right)=A_{n}=\left\{\hat{x}:\;\hat{H}\left(\hat{x}\right)=H_{0}\right\}.$$

Therefore, every sequence whose empirical distribution pertains to empirical entropy different from $H_{0}$ should be rejected. To this end, our discriminator should be able to gather empirical statistics, namely to count occurrences of symbols (or, more generally, count combinations of symbols and states) and not just detect a forbidden pattern that might have occurred just once in ***x***.

This motivates us to broaden the class of finite-state discriminators to be considered in the following way. We consider discriminators that consist of a next-state function,

(17) $$z_{i+1}=g\left(z_{i},{\hat{x}}_{i}\right),$$

as before, but instead of the binary output function, *f*, as in [25], these discriminators are equipped with a set of α·s counters that count the number of joint occurrences of all (x,z)∈X×S for i=0,1,2,…,n−1, i.e.,

(18) $$n\left(x,z\right)=\sum_{i=0}^{n-1}I\left\{{\hat{x}}_{i}=x,\;z_{i}=z\right\},\;\;\;\;\;x\in X,\;z\in S,$$

where I{A}, for a generic event *A*, denotes its indicator function, namely I{A}=1 if *A* is true; otherwise, I{A}=0. A sequence $\hat{x}$ is accepted (resp. rejected) if the matrix of counts, {n(x,z),x∈X,z∈S}, satisfies (resp. violates) a certain condition. In the example in the previous paragraph, a sequence is accepted if

(19) $$\hat{H}\left(\hat{x}\right)=\sum_{x\in X}\frac{n(x)}{n}\log\left(\frac{n}{n(x)}\right)=H_{0},$$

where $n\left(x\right)=\sum_{z\in S}n\left(x,z\right)$. Ziv’s discriminator model is clearly a special case of this model: let $\left(x_{★},z_{★}\right)$ be any combination of input and state such that $f(z_{★},x_{★})=1$ in Ziv’s model (that is, a “forbidden” combination). Then, in terms of the proposed extended model, a sequence is rejected whenever $n(x_{★},z_{★})\geq 1$.

Clearly, since $x\in A_{n}$ and since membership in $A_{n}$ depends solely on the matrix of counts, {n(x,z),x∈X,z∈S}, it follows that all $\left\{\hat{x}\right\}$ that share the same counts as those of ***x*** must also be members of $A_{n}$. The set of all $\left\{\hat{x}\right\}$ of length *n* with the same counts, {n(x,z),x∈X,z∈S}, as those of ***x*** is called the *finite-state type class* with respect to the FSM *g* (see also [3]), and it is denoted by $T_{g}\left(x\right)$. Since $A_{n}⊇T_{g}\left(x\right)$,

(20) $$|A_{n}\left|\geq \right|T_{g}\left(x\right)|.$$

It was proven in [3] (in Lemma 3), that if n(x,z)≥n(z)δ(n) for every (x,z)∈X×S, where δ(n)>0 may even be a vanishing sequence, then

(21) $$|T_{g}\left(x\right)|\geq \exp_{2}\left\{n\hat{H}\left(X|Z\right)-\frac{s(\alpha -1)}{2}\cdot \log\left(2\pi n\right)\right\},$$

where

(22) $$\hat{H}\left(X|Z\right)=-\sum_{x\in X}\sum_{z\in S}\frac{n(x,z)}{n}\log\frac{n(x,z)}{n(z)},$$

with $n\left(z\right)=\sum_{x\in X}n\left(x,z\right)$ and with the conventions that $0\log0\overset{▵}{=}0$ and $0/0\overset{▵}{=}0$. Therefore, it follows that the key rate needed for perfect secrecy is lower bounded by

(23) $$\begin{array}{cc} R & =\frac{H(K)}{n}\\ & \geq \frac{\log|A_{n}|}{n}\\ & \geq \frac{\log|T_{g}\left(x\right)|}{n}\\ & \geq \hat{H}\left(X|Z\right)-\frac{s(\alpha -1)}{2}\cdot \frac{\log(2\pi n)}{n}. \end{array}$$

This lower bound can be asymptotically attained through an encrypter that applies universal lossless compression for finite-state sources with the next-state function *g*, followed by one-time pad encryption. This universal lossless compression scheme is based on a conceptually simple extension of the above-mentioned universal scheme for the class of memoryless sources: one applies a two-part code, where the first part includes a code for the index of the type class with respect to *g* (using a number of bits that is proportional to log n), and the second part encodes the index of the location of $\hat{x}$ within $T_{g}\left(x\right)$ according to a predefined order agreed upon between the encoder and the decoder (see [3] for more details). More precisely, the compression ratio that can be achieved, which is also the key rate consumed, is upper bounded by (see Section III in [3]):

(24) $$R\leq \hat{H}\left(X|Z\right)+\frac{s(\alpha -1)}{2}\cdot \frac{\log\;n}{n}+O\left(\frac{1}{n}\right),$$

which tells us that the gap between the lower bound and the achievability is proportional to $\frac{\log\;n}{n}$, which decays much faster than the aforementioned $O\left(\frac{\log(\log\;n)}{\log\;n}\right)$ convergence rate of the gap in Ziv’s approach. Moreover, for most sequences in most type classes, $\left\{T_{g}\left(x\right)\right\}$, the coding rate (which is also the key rate in one-time pad encryption) of $\hat{H}\left(X|Z\right)+\frac{s(\alpha -1)}{2}\cdot \frac{\log\;n}{n}$ is smaller than LZ(x)/n since the former quantity is essentially also a lower bound to the compression ratio of any lossless compression scheme for most individual sequences in $T_{g}\left(x\right)$ for almost all such type classes (see Theorem 1 in [3]). The converse inequality between $\hat{H}\left(X|Z\right)$ and LZ(x)/n, which follows from Ziv’s inequality (see [28,29] (Lemma 13.5.5 in [28])) holds up to an $O\left(\frac{\log(\log\;n)}{\log\;n}\right)$ term again.

**Remark** **3.** *To put Ziv’s result in perspective, one may wish to envision a class of discriminators defined by a given dictionary of c distinct ‘words’, which are the c phrases in Ziv’s derivation [25] (see also Appendix A). A possible definition of such a discriminator is that it accepts only n-sequences of plaintext that are formed by concatenating words from the given dictionary, allowing repetitions. These are no longer finite-state discriminators. In this case, Ziv’s derivation is applicable under the minor modification that the various phrases should be classified only in terms of their length but without further partitioning according to initial and final states (z and $z^{\prime }$ in the derivation in Appendix A). This is equivalent to assuming that s=1, and accordingly, the term $\frac{2c\log\;s}{n}$ in the last equation in Appendix A should be omitted. Of course, the resulting bound can still be matched through LZ78 compression, followed by one-time pad encryption.*

### 3.2. Shift-Register Machines with Counters

Since the eavesdropper naturally does not cooperate with the encrypter, the latter might not know the particular FSM, *g*, used by the former, and therefore, it is instructive to derive key-rate bounds that are independent of *g*. To this end, consider the following. Given ***x*** and a fixed positive integer *ℓ* (ℓ≪n), let us observe the more refined joint empirical distribution,

(25) $${\hat{P}}_{X^{\ell +1}Z^{\ell +1}}\left(a_{0},\ldots ,a_{\ell },s_{0},\ldots ,s_{\ell }\right)=\frac{1}{n}\sum_{i=0}^{n-1}I\left\{x_{i}=a_{0},\ldots ,x_{i⊕\ell }=a_{\ell },z_{i}=s_{0},\ldots ,z_{i⊕\ell }=s_{\ell }\right\},$$

as well as all partial marginalizations derived from this distribution, where ⊕ denotes addition modulo *n* so as to create a periodic extension of ***x*** (hence, redefining $z_{0}=g\left(z_{n-1},x_{n-1}\right)$). Accordingly, the previously defined empirical conditional entropy, $\hat{H}\left(X|Z\right)$, is now denoted as $\hat{H}\left(X_{1}|Z_{1}\right)$, which is also equal to $\hat{H}\left(X_{2}|Z_{2}\right)$, etc., due to the inherent shift-invariance property of the empirical joint distribution extracted under the periodic extension of ***x***. Consider now the following chain of inequalities:

$$\begin{array}{ccc} & & \hat{H}\left(X_{\ell }|X_{0},X_{1},\ldots ,X_{\ell -1}\right)-\hat{H}\left(X_{\ell }|Z_{\ell }\right) \end{array}$$

(26) $$\begin{array}{ccc} & \leq & \frac{1}{\ell +1}\sum_{j=0}^{\ell }\left[\hat{H}\left(X_{j}|X_{0},\ldots ,X_{j-1}\right)-\hat{H}\left(X_{j}|X_{0},\ldots ,X_{j-1},Z_{0}\right)\right] \end{array}$$

(27) $$\begin{array}{ccc} & = & \frac{1}{\ell +1}\left[\hat{H}\left(X_{0},\ldots ,X_{\ell }\right)-\hat{H}\left(X_{0},\ldots ,X_{\ell }|Z_{0}\right)\right] \end{array}$$

(28) $$\begin{array}{ccc} & = & \frac{\hat{I}\left(Z_{0};X_{0},\ldots ,X_{\ell }\right)}{\ell +1} \end{array}$$

(29) $$\begin{array}{ccc} & \leq & \frac{\hat{H}\left(Z_{0}\right)}{\ell +1} \end{array}$$

(30) $$\begin{array}{ccc} & \leq & \frac{\log s}{\ell +1}, \end{array}$$

where $\hat{I}\left(\cdot ;\cdot \right)$ denotes empirical mutual information, and where in the second line, the term corresponding to j=0 should be understood to be $\left[\hat{H}\left(X_{0}\right)-\hat{H}\left(X_{0}|Z_{0}\right)\right]$. The first inequality follows because given $\left(X_{0},\ldots ,X_{j-1},Z_{0}\right)$, one can reconstruct $Z_{1},Z_{2},\ldots ,Z_{j}$ through *j* recursive applications of the next-state function, *g*. Therefore,

$$\begin{array}{ccc} \hat{H}\left(X_{j}|X_{0},\ldots ,X_{j-1},Z_{0}\right) & = & \hat{H}\left(X_{j}|X_{0},\ldots ,X_{j-1},Z_{0},Z_{1},\ldots ,Z_{j}\right) \end{array}$$

(31) $$\begin{array}{ccc} & \leq & \hat{H}\left(X_{j}|Z_{j}\right) \end{array}$$

(32) $$\begin{array}{ccc} & = & \hat{H}\left(X_{\ell }|Z_{\ell }\right). \end{array}$$

Equivalently,

(33) $$\hat{H}\left(X_{\ell }|Z_{\ell }\right)\geq \hat{H}\left(X_{\ell }|X_{0},\ldots ,X_{\ell -1}\right)-\frac{\log s}{\ell +1},$$

and by combining this with Equations (20) and (21), we obtain

(34) $$\log|A_{n}|\geq n\left[\hat{H}\left(X_{\ell }|X_{0},\ldots ,X_{\ell -1}\right)-\frac{\log s}{\ell +1}\right]-\frac{s(\alpha -1)}{2}\cdot \log\left(2\pi n\right).$$

The advantage of this inequality is that it is independent of the arbitrary next-state function *g*. In fact, we actually replaced the arbitrary FSM, *g*, with a particular FSM—the shift-register FSM—whose state is $z_{i}=\left(x_{i-\ell },x_{i-\ell +1},\ldots ,x_{i-1}\right)$ at the cost of a gap of $\frac{\log s}{\ell +1}$, which can be kept arbitrarily small if the size of the shift-register, *ℓ*, is sufficiently large compared to the memory size, logs, of *g*.

**Remark** **4.** *The fact that $\hat{H}\left(X_{\ell }|X_{0},\ldots ,X_{\ell -1}\right)$ cannot be much larger than $\hat{H}\left(X_{\ell }|Z_{\ell }\right)$ for large enough ℓ actually suggests that whatever the state of any FSM g can possibly “remember” from the past of **x** is essentially captured by the recent past, not by the remote past. While this is not surprising in the context of the probabilistic setting, especially if the underlying random process is ergodic and hence has a vanishing memory of the remote past, this finding is not quite trivial when it comes to arbitrary individual sequences.*

Returning to our derivations, in view of the first three lines of (13), the key rate needed for perfect secrecy is lower bounded by

(35) $$R\geq \frac{\log|A_{n}|}{n}\geq \hat{H}\left(X_{\ell }|X_{0},\ldots ,X_{\ell -1}\right)-\frac{\log s}{\ell +1}-\frac{s(\alpha -1)}{2n}\cdot \log\left(2\pi n\right),$$

and since this holds for any *ℓ* in some fixed range 1≤ℓ≤l (i.e., where *l* is independent of *n*),

(36) $$R\geq \max_{1\leq \ell \leq l}\left[\hat{H}\left(X_{\ell }|X_{0},\ldots ,X_{\ell -1}\right)-\frac{\log s}{\ell +1}\right]-\frac{s(\alpha -1)}{2n}\cdot \log\left(2\pi n\right).$$

Note that if ***x*** is an “$\ell_{0}$-th order Markovian sequence” in the sense that $\hat{H}\left(X_{\ell }|X_{0},\ldots ,X_{\ell -1}\right)$ is almost fixed for all $\ell_{0}\leq \ell \leq l$ with l≫logs, then $\ell_{0}$ is the preferred choice for *ℓ* as it essentially captures the best attainable key rate.

This lower bound can be asymptotically attained through universal lossless compression for *ℓ*-th order Markov types [30,31,32] (see Section VII.A in [32]), followed by one-time pad encryption, where the achieved rate is

(37) $$R\leq \hat{H}\left(X_{\ell }|X_{0},\ldots ,X_{\ell -1}\right)+\frac{\alpha^{\ell -1}\left(\alpha -1\right)}{2}\cdot \frac{\log\;n}{n}+O\left(\frac{1}{n}\right).$$

In this case, $A_{n}$ is the *ℓ*-th order Markov type of ***x***, and the finite-state discriminator of the eavesdropper is a shift-register machine, which checks whether the *ℓ*-th order Markov type of each $\hat{x}$ has the matching empirical conditional entropy of order *ℓ*.

In this result, there is compatibility between the converse bound and the achievability bound in the sense that both are given in terms of FSMs with a fixed number of states that do not grow with *n*. Among all possible finite-state machines, we have actually singled out the shift-register machine universally with a controllable asymptotic gap of $\frac{\log s}{\ell +1}$. However, the bound is otherwise explicit, and it is clear how to approach it. If we wish to keep this gap below a given ϵ>0, we select ℓ=⌈(logs)/ϵ⌉−1. In this sense, it is another refinement of Ziv’s result.

### 3.3. Periodically Time-Varying FSMs with Counters

So far, we have considered time-invariant FSMs, where the function *g* remains fixed over time. We now expand the scope to consider the class of discriminators implementable by periodically time-varying FSMs, defined as follows:

(38) $$\begin{array}{ccc} z_{i+1} & = & g(z_{i},{\hat{x}}_{i},i\;\mathrm{mod}\;l), \end{array}$$

where i=0,1,2,… and *l* is a positive integer that designates the period of the time-varying finite-state machine. Conceptually, this is not really more general than the ordinary, time-invariant FSM defined earlier because the state of the modulo-*ℓ* clock can be considered part of the entire state. In other words, this is a time-invariant FSM with s·l states, indexed by the ordered pair $\left(z_{i},i\;mod\;l\right)$. The reason it makes sense to distinguish between the state $z_{t}$ and the state of the clock is that the clock does not store any information regarding past input data. This is to say that in the context of time-varying finite-state machines, we distinguish between the amount of memory of past input (logs bits) and the period *l*. Both parameters manifest the richness of the class of machines but in different manners. Indeed, the parameters *s* and *l* play very different and separate roles in the converse bound derived below.

**Remark** **5.** *Clearly, the earlier considered time-invariant finite-state machine is obtained as a special case through l=1, or through the case where l is arbitrary, but the next-state functions, g(·,·,0),g(·,·,1),…,g(·,·,l−1), are all identical.*

First, observe that a periodic FSM with period *l* can be viewed as a time-invariant FSM at the level of *l*-blocks, $\left\{x_{il},x_{il+1},\ldots ,x_{il+l-1}\right\}$, i=0,1,…, and hence also at the level of *ℓ*-blocks, where *ℓ* is an integer multiple of *l*. Accordingly, let *ℓ* be an arbitrary integer multiple of *l*, but at the same time, assume that *ℓ* divides *n*. Denote m=n/ℓ, and define the counts,

(39) $$m\left(z,z^{\prime },x^{\ell }\right)=\sum_{i=0}^{n/\ell -1}I\left\{z_{i\ell }=z,\;z_{i\ell +\ell }=z^{\prime },\;{\hat{x}}_{i\ell }^{i\ell +\ell -1}=x^{\ell }\right\},\;\;\;\;z,z^{\prime }\in S,\;x^{\ell }\in X^{\ell }.$$

In fact, there is a certain redundancy in this definition because $z^{\prime }$ is a deterministic function of $\left(z,x^{\ell }\right)$ obtained through *ℓ* recursive applications of the (time-varying) next-state function *g*. Concretely, $m\left(z,z^{\prime },x^{\ell }\right)=m\left(z,x^{\ell }\right)$ if $z^{\prime }$ matches $\left(z,x^{\ell }\right)$ and $m(z,z^{\prime },x^{\ell })=0$ otherwise. Nonetheless, we adopt this definition for the sake of clarity of combinatorial derivation to be carried out shortly. In particular, our derivation is based on grouping together all $\left\{x^{\ell }\right\}$, which, for a given *z*, yields the same $z^{\prime }$. Accordingly, we also denote $m\left(z,z^{\prime }\right)=\sum_{x^{\ell }\in X^{\ell }}m\left(z,z^{\prime },x^{\ell }\right)$.

Suppose that the acceptance/rejection criterion that defines $A_{n}$ is based on the counts, $\left\{m\left(z,z^{\prime },x^{\ell }\right),\;z,z^{\prime }\in S,\;x^{\ell }\in X^{\ell }\right\}$, and then the smallest $A_{n}$ that contains ***x*** is the type class of ***x*** pertaining to $\left\{m\left(z,z^{\prime },x^{\ell }\right),\;z,z^{\prime }\in S,\;x^{\ell }\in X^{\ell }\right\}$. The various sequences in this type class are obtained by permuting distinct *ℓ*-tuples $\left\{{\hat{x}}_{i\ell }^{i\ell +\ell -1},\;i=0,1,\ldots ,m-1\right\}$ that begin at the same state, *z*, and end at the same state, $z^{\prime }$. Let us define the empirical distribution as

(40) $$\hat{P}\left(z,z^{\prime },x^{\ell }\right)=\frac{m(z,z^{\prime },x^{\ell })}{m},\;\;\;\;z,z^{\prime }\in S,\;a^{\ell }\in X^{\ell },$$

and the joint entropy as

(41) $$\hat{H}\left(Z,Z^{\prime },X^{\ell }\right)=-\sum_{z,z^{\prime },x^{\ell }}\hat{P}\left(z,z^{\prime },x^{\ell }\right)\log\hat{P}\left(z,z^{\prime },x^{\ell }\right),$$

and let

(42) $$\hat{H}\left(X^{\ell }|Z,Z^{\prime }\right)=\hat{H}\left(Z,Z^{\prime },X^{\ell }\right)-\hat{H}\left(Z,Z^{\prime }\right),$$

where $\hat{H}\left(Z,Z^{\prime }\right)$ is the marginal empirical entropy of $\left(Z,Z^{\prime }\right)$. Then, using the method of types [32], we have:

(43) $$\begin{array}{ccc} |A_{n}| & \geq & \prod_{z,z^{\prime }\in S}\frac{m(z,z^{\prime })!}{\prod_{x^{\ell }\in X^{\ell }}m\left(z,z^{\prime },x^{\ell }\right)!} \end{array}$$

(44) $$\begin{array}{ccc} & \geq & \prod_{z,z^{\prime }\in S}\left[{\left(m+1\right)}^{-\alpha^{\ell }}\cdot \exp_{2}\left\{\sum_{x^{\ell }}m\left(z,z^{\prime },x^{\ell }\right)\log\frac{m(z,z^{\prime })}{m(z,z^{\prime },x^{\ell })}\right\}\right] \end{array}$$

(45) $$\begin{array}{ccc} & \geq & {\left(m+1\right)}^{-s^{2}\alpha^{\ell }}\cdot 2^{m\hat{H}\left(X^{\ell }|Z,Z^{\prime }\right)} \end{array}$$

(46) $$\begin{array}{ccc} & = & {\left(m+1\right)}^{-s^{2}\alpha^{\ell }}\cdot 2^{m[\hat{H}\left(X^{\ell }\right)-I\left(Z,Z^{\prime };X^{\ell }\right)]} \end{array}$$

(47) $$\begin{array}{ccc} & \geq & {\left(m+1\right)}^{-s^{2}\alpha^{\ell }}\cdot 2^{m[\hat{H}\left(X^{\ell }\right)-H\left(Z,Z^{\prime }\right)]} \end{array}$$

(48) $$\begin{array}{ccc} & \geq & {\left(m+1\right)}^{-s^{2}\alpha^{\ell }}\cdot 2^{m[\hat{H}\left(X^{\ell }\right)-2\log s]} \end{array}$$

(49) $$\begin{array}{ccc} & = & \exp_{2}\left\{n\left[\frac{\hat{H}\left(X^{\ell }\right)}{\ell }-\frac{2\log s}{\ell }-\frac{s^{2}\alpha^{\ell }}{n}\log\left(\frac{n}{\ell }+1\right)\right]\right\}, \end{array}$$

and so

(50) $$R\geq \frac{\log|A_{n}|}{n}\geq \frac{\hat{H}\left(X^{\ell }\right)}{\ell }-\frac{2\log s}{\ell }-\frac{s^{2}\alpha^{\ell }}{n}\log\left(\frac{n}{\ell }+1\right),$$

which, for ℓ≫2logs, can be essentially matched through universal compression for block-memoryless sources and one-time pad encryption using exactly the same ideas as before. Once again, we have derived a lower bound that is free of dependence on the particular FSM, *g*. Recall that *ℓ* divides *n* and that it is also a multiple of *l*, but otherwise, *ℓ* is arbitrary. Hence we may maximize this lower bound with respect to *ℓ* subject to these constraints. Alternatively, we may rewrite the lower bound as

(51) $$R\geq \max_{\left\{q\;divides\;n/l\right\}}\left\{\frac{\hat{H}\left(X^{ql}\right)}{ql}-\frac{2\log s}{ql}-\frac{s^{2}\alpha^{ql}}{n}\log\left(\frac{n}{ql}+1\right)\right\}.$$

Clearly, in view of Remark 5, the above lower bound also applies to the case of a time-invariant FSM, *g*, but then there would be some mismatch between the upper and lower bounds because to achieve the lower bound, one must gather more detailed empirical statistics, namely empirical statistics of blocks together with states, rather than just single symbols with states.

## 4. Side Information

Some of the results presented in the previous sections extend to the case where side information (SI) is available to both the legitimate decrypter and the eavesdropper. In principle, it may or may not be available to the encrypter, and we consider first the case where it is available. We assume the SI sequence to be of length *n*, denoted by $y=(y_{0},y_{1},\ldots ,y_{n-1})$, where $y_{i}\in Y$, i=0,1,…,n−1. It is related to the plaintext, ***x***, to be encrypted, but like ***x***, it is a deterministic, individual sequence. The SI alphabet Y is finite, and its cardinality, |Y|, is denoted by β. Here, the acceptance set depends on ***y*** and is denoted accordingly by $A_{n}\left(y\right)$. The encrypter is, therefore, a mapping W=T(x,y,K), which is invertible given (y,K), allowing the decrypter to reconstruct $x=T^{-1}\left(W,y,K\right)$.

Consider first a natural extension of Ziv’s model for a finite-state discriminator, which for i=0,1,…,n−1, implements the recursion,

(52) $$\begin{array}{ccc} u_{i} & = & f(z_{i},{\hat{x}}_{i},y_{i}) \end{array}$$

(53) $$\begin{array}{ccc} z_{i+1} & = & g(z_{i},{\hat{x}}_{i},y_{i}). \end{array}$$

Similarly as before, the acceptance set, $A_{n}\left(y\right)$ is the set of all {x} that, in the presence of the given ***y***, yield the all-zero output sequence, $u=\left(u_{0},u_{1},\ldots ,u_{n-1}\right)=\left(0,0,\ldots ,0\right)$.

Let c(x,y) denote the number of joint parsing phrases of

$$\left(x,y\right)=(\left(x_{0},y_{0}\right),\left(x_{1},y_{1}\right),\ldots ,\left(x_{n-1},y_{n-1}\right))$$

into distinct phrases, and let $c_{l}\left(x|y\right)$ denote the number of occurrences of y(l)—the *l*-th distinct phrase of ***y***—which is also the number of different phrases of ***x*** that appear jointly with y(l). Let c(y) denote the number of different {y(l)}, that is, $\sum_{l=1}^{c(y)}c_{l}\left(x|y\right)=c\left(x,y\right)$. Finally, let $c_{lzz^{\prime }}\left(x|y\right)$ denote the number of ***x***-phrases that are aligned with y(l), beginning at state *z* and ending at state $z^{\prime }$. Then, similarly as in the derivation in [25] (see also Appendix A),

(54) $$|A_{n}\left(y\right)|\geq \prod_{l=1}^{c(y)}\prod_{z,z^{\prime }}c_{lzz^{\prime }}{\left(x|y\right)}^{c_{lzz^{\prime }}\left(x|y\right)},$$

and so, defining the auxiliary RVs $\left(Z_{l},Z_{l}^{\prime }\right)$, l=1,…,c(y), as being jointly distributed according to

(55) $$Q_{l}\left(z,z^{\prime }\right)=\frac{c_{lzz^{\prime }}\left(x|y\right)}{c_{l}\left(x|y\right)},\;\;\;z,z\in S,$$

we have

(56) $$\begin{array}{cc} \log|A_{n}\left(y\right)| & \geq \sum_{l=1}^{c(y)}\sum_{z,z^{\prime }}c_{lzz^{\prime }}\left(x|y\right)\log c_{lzz^{\prime }}\left(x|y\right)\\ & =\sum_{l=1}^{c(y)}c_{l}\left(x|y\right)\sum_{z,z^{\prime }}\frac{c_{lzz^{\prime }}\left(x|y\right)}{c_{l}\left(x|y\right)}\left[\log\frac{c_{lzz^{\prime }}\left(x|y\right)}{c_{l}\left(x|y\right)}+\log c_{l}\left(x|y\right)\right]\\ & =\sum_{l=1}^{c(y)}c_{l}\left(x|y\right)\log c_{l}\left(x|y\right)-\sum_{l=1}^{c(y)}c_{l}\left(x,y\right)H\left(Z_{l},Z_{l}^{\prime }\right)\\ & \geq \sum_{l=1}^{c(y)}c_{l}\left(x|y\right)\log c_{l}\left(x|y\right)-2\cdot \sum_{l=1}^{c(y)}c_{l}\left(x,y\right)\log s\\ & =\sum_{l=1}^{c(y)}c_{l}\left(x|y\right)\log c_{l}\left(x|y\right)-2c\left(x,y\right)\log s\\ & \geq \sum_{l=1}^{c(y)}c_{l}\left(x|y\right)\log c_{l}\left(x|y\right)-\frac{2n\log s}{(1-\epsilon_{n})\log n}, \end{array}$$

where the last inequality follows from [7] (see also Lemma 13.5.3 in [28]), with $\epsilon_{n}=O\left(\frac{\log(\log\;n)}{\log\;n}\right)$. This lower bound can be asymptotically attained through the conditional version of the LZ algorithm (see [33,34]), followed by one-time pad encryption. If ***y*** is unavailable to the encrypter, ***x*** can still be compressed into about

(57) $$u\left(x|y\right)=\sum_{l=1}^{c(y)}c_{l}\left(x|y\right)\log c_{l}\left(x|y\right)$$

bits before one-time pad encryption, using Slepian–Wolf encoding (Section 15.4 in [28]) and reconstructing (with high probability) after decryption using a universal decoder that uses u(x|y) as a decoding metric (see [35]).

Unfortunately, and somewhat surprisingly, a direct extension of the results in Section 3.1 and Section 3.2 to the case with SI turns out to be rather elusive. The reason is the lack of a single-letter formula for the exponential growth rate of the cardinality of a finite-state conditional type class of *x*-vectors that is defined by joint counts of the form $n\left(x,y,z\right)=\sum_{i=0}^{n-1}I\left\{x_{i}=x,y_{i}=y,z_{i}=z\right\}$, even in the simplest special case where $z_{i}=x_{i-1}$. In a nutshell, this quantity depends on ***y*** in a complicated manner, which cannot be represented in terms of an empirical distribution of fixed dimension that does not grow with *n*. However, we can obtain at least a lower bound if we treat these cases as special cases of periodically time-varying FSMs with counters in view of Remark 5.

Finally, consider the class of discriminators implementable by periodically time-varying FSMs with SI, defined as follows:

(58) $$\begin{array}{ccc} z_{i+1} & = & g(z_{i},{\hat{x}}_{i},y_{i},i\;\mathrm{mod}\;l), \end{array}$$

where i=0,1,2,… and *l* is as in Section 3.3. The extension of the derivation in Section 3.3 is quite straightforward—one has to count the permutations of the *ℓ*-vectors of ***x*** that not only share the same initial and final states but are also aligned to the same *ℓ*-blocks of ***y***. The resulting bound is given by:

(59) $$R\geq \frac{\log|A_{n}\left(y\right)|}{n}\geq \frac{\hat{H}\left(X^{\ell }|Y^{\ell }\right)}{\ell }-\frac{2\log s}{\ell }-\frac{s^{2}\alpha^{\ell }\beta^{\ell }}{n}\log\left(\frac{n}{\ell }+1\right),$$

which, for ℓ≫2logs, can be essentially matched through universal compression for block-memoryless sources in the presence of SI and one-time pad encryption. In the absence of SI at the encrypter, one may use universal Slepian–Wolf coding for block-memoryless sources, which is a direct extension of universal Slepian–Wolf coding for memoryless sources (see, e.g., [36]).

## Data Availability

Not applicable.

## Appendix A. Proof of the Last Step of Equation (13)

Since [25] is unpublished, for the sake of completeness, we provide here the essential steps of Ziv’s proof of the inequality,

(A1) $$\log|A_{n}|\geq LZ\left(x\right)-n\epsilon_{n},$$

which must hold for every finite-state discriminator with no more than *s* states, where $\epsilon_{n}=O\left(\frac{\log(\log n)}{\log n}\right)$ for fixed *s*. The final steps are different from those in [25], as we adopt a somewhat simpler approach, which is presented in Section 13.5 in [28].

Let ***x*** be parsed into *c* distinct phrases,

$$\left(x_{0},x_{1},\ldots ,x_{n_{1}-1}\right),\left(x_{n_{1}},x_{n_{1}+1},\ldots ,x_{n_{2}-1}\right),\ldots ,\left(x_{n_{c}},x_{n_{c}+1},\ldots ,x_{n-1}\right),$$

with the possible exception of the last phrase, which might be incomplete. Let $c_{lzz^{\prime }}$ denote the number of phrases of length *l*, where the initial state of the FSM *g* is *z* and the final state is $z^{\prime }$ (of course, $\sum_{l,z,z^{\prime }}c_{lzz^{\prime }}=c$). If the discriminator accepts ***x***, namely if u=(0,0,…,0), then for every $\left(l,z,z^{\prime }\right)$, each of the $c_{lzz^{\prime }}$ phrases can be replaced with any of the other such phrases to obtain new sequences of length *n* for which the output is also u=(0,0,…,0), and hence must be accepted too. Now, let $\left(L,Z,Z^{\prime }\right)$ denote a triple of auxiliary random variables jointly distributed according to the probability distribution

(A2) $$Q\left(l,z,z^{\prime }\right)=\frac{c_{lzz^{\prime }}}{c},\;\;\;\;\;\;z,z^{\prime }\in S,\;l=1,2,\ldots$$

and let $H(L,Z,Z^{\prime })$ denote the joint entropy of $\left(L,Z,Z^{\prime }\right)$. Then,

(A3) $$|A_{n}|\geq \prod_{l,z,z^{\prime }}{\left(c_{lzz^{\prime }}\right)}^{c_{lzz^{\prime }}},$$

where ${\left(c_{lzz^{\prime }}\right)}^{c_{lzz^{\prime }}}$ on the right-hand side is the number of ways each one of the $c_{lzz^{\prime }}$ phrases of length *l*, starting at state *z* and ending at state $z^{\prime }$, can be replaced with any other phrase with the same qualifiers. Consequently,

(A4) $$\begin{array}{ccc} \log|A_{n}| & \geq & \sum_{l,z,z^{\prime }}c_{lzz^{\prime }}\log c_{lzz^{\prime }} \end{array}$$

(A5) $$\begin{array}{ccc} & = & c\cdot \sum_{l,z,z^{\prime }}\frac{c_{lzz^{\prime }}}{c}\left[\log\frac{c_{lzz^{\prime }}}{c}+\log c\right] \end{array}$$

(A6) $$\begin{array}{ccc} & = & c\log c-c\cdot H(L,Z,Z^{\prime }) \end{array}$$

(A7) $$\begin{array}{ccc} & \geq & c\log c-c[H\left(L\right)+H\left(Z\right)+H\left(Z^{\prime }\right)] \end{array}$$

(A8) $$\begin{array}{ccc} & \geq & c\log c-c\cdot H(L)-2c\log s. \end{array}$$

Now, the entropy of *L*, given that E{L}=n/c, cannot be larger than (n/c+1)log(n/c+1)−(n/c)log(n/c)≤1+log(n/c+1) (see Lemma 13.5.4 and Equations (13.120)–(13.122) in [28]). Thus,

(A9) $$\log|A_{n}|\geq c\log c-2c\log s-c-c\log\left(\frac{n}{c}+1\right),$$

and then

(A10) $$\begin{array}{cc} R & \geq \frac{\log|A_{n}|}{n}\\ & \geq \frac{c\log c}{n}-\frac{2c}{n}\log s-\frac{c}{n}-\frac{c}{n}\log\left(\frac{n}{c}+1\right)\\ & =\frac{c\log c}{n}-\frac{2c}{n}\log s-\frac{c}{n}-O\left(\frac{\log\log n}{\log n}\right), \end{array}$$

where the last line is derived similarly as in Equations (13.123)–(13.124) in [28]. The final step of Equation (13) is obtained from the fact that LZ(x) is upper bounded by clog c plus terms that, after normalizing by *n*, are negligible compared to $\frac{\log(\log\;n)}{\log\;n}$ (see Theorem 2 in [7]).
